# Involvement of gap junction channels in the pathophysiology of migraine with aura

**DOI:** 10.3389/fphys.2014.00078

**Published:** 2014-02-25

**Authors:** Denis Sarrouilhe, Catherine Dejean, Marc Mesnil

**Affiliations:** ^1^Laboratoire de Physiologie Humaine, Faculté de Médecine et Pharmacie, Université de PoitiersPoitiers, France; ^2^Service Pharmacie, Centre Hospitalier Henri LaboritPoitiers, France; ^3^Faculté des Sciences Fondamentales et Appliquées, STIM, ERL 7368-CNRS, Université de PoitiersPoitiers, France

**Keywords:** aura, connexin, cortical spreading depression, gap junction, pannexin, tonabersat, trigeminovascular

## Abstract

Migraine is a common, recurrent, and disabling primary headache disorder with a genetic component which affects up to 20% of the population. One third of all patients with migraine experiences aura, a focal neurological disturbance that manifests itself as visual, sensitive or motor symptoms preceding the headache. In the pathophysiology of migraine with aura, activation of the trigeminovascular system from the meningeal vessels mediates migraine pain via the brainstem and projections ascend to the thalamus and cortex. Cortical spreading depression (CSD) was proposed to trigger migraine aura and to activate perivascular trigeminal nerves in the cortex. Quinine, quinidine and the derivative mefloquine are able to inhibit CSD suggesting an involvement of neuronal connexin36 channels in CSD propagation. More recently, CSD was shown to induce headache by activating the trigeminovascular system through the opening of stressed neuronal Pannexin1 channels. A novel benzopyran compound, tonabersat, was selected for clinical trial on the basis of its inhibitory activity on CSD and neurogenic inflammation in animal models of migraine. Interestingly, in the time course of animal model trials, tonabersat was shown to inhibit trigeminal ganglion (TGG) neuronal-glial cell gap junctions, suggesting that this compound could prevent peripheral sensitization within the ganglion. Three clinical trials aimed at investigating the effectiveness of tonabersat as a preventive drug were negative, and conflicting results were obtained in other trials concerning its ability to relieve attacks. In contrast, in another clinical trial, tonabersat showed a preventive effect on attacks of migraine with aura but had no efficacy on non-aura attacks. Gap junction channels seem to be involved in several ways in the pathophysiology of migraine with aura and emerge as a new promising putative target in treatment of this disorder.

## Introduction

Migraine is a common, recurrent, and disabling primary headache disorder which affects up to 20% of the population (Haut et al., 2006). The estimated annual prevalence of migraine is 18.2% in women and 6.5% in men in the population of the United States, and 16.8 and 7.5%, respectively, in European countries (Lipton et al., 2001; Stovner et al., 2006). Migraine prevalence varies with age and is highest in 35- to 45-year-old people. Migraine is associated with significant reduction of health-related quality of life and has an important socioeconomic impact. Migraineurs report functional impairment with their headaches: bed rest, absenteeism from work or school, reduced effectiveness, disruption of household work, family or social activity. On the basis of clinical and descriptive criteria, and headache features, the second edition of the Classification of Headache Disorders divided migraine into five major categories, the two most important of these are migraine without aura and migraine with aura. About one third of all patients with migraine experiences aura, a focal neurological disturbance that manifests itself as visual, sensitive or motor symptoms preceding the headache. The classification also acknowledges rare forms of this disease as familial hemiplegic migraine, the first migraine syndrome to be linked to a specific set of genetic polymorphisms (Lipton et al., 2004). Whereas there are evidences for a genetic contribution to migraine and that environmental factors also play a role, the brain events that initiate migraine remains unclear. This disease is underdiagnosed and also undertreated. Traditionally, treatment for migraine is divided into acute and preventive approaches, aimed at stopping the evolving attack or stopping the onset of attacks, respectively. Drugs used in acute treatment target the serotonergic system (triptans and ergot derivatives), the inflammatory reaction [non-steroidal anti-inflammatory drugs (NSAIDs), aspirin] or calcitonin gene-related peptide (CGRP) receptors (gepants). Some preventive drugs target the central nervous system (CNS) inhibitory [gamma-aminobutyric acid (GABA) receptors] and excitatory systems (glutamatergic neurotransmission, ion channels) and belong to the family of antiepileptic drugs. α1 blockers (oxetorone, indoramine) and β1 blockers (propranolol, metoprolol) can also be used in migraine prophylaxis.

Recent studies show that gap junction channels seem to be involved in several ways in the pathophysiology of migraine with aura (Sarrouilhe and Dejean, 2012; Karatas et al., 2013). A novel benzopyran compound, (-)-*cis*-6-acetyl-4*S*-(3-chloro-4-fluoro-benzoylamino)-3,4-dihydro-2,2-dimethyl-2*H*-benzo[b]pyrane-3*S*-ol (SB-220453, tonabersat), was selected for clinical trials as an anti-migraine agent. Preclinical studies showed that tonabersat inhibited cortical spreading depression (CSD) and neurogenic inflammation in animal models of migraine (Durham and Garrett, 2009). CSD is a wave of electrical activity that moves across the cerebral cortex and was proposed to trigger migraine aura and to induce migraine headache (Charles and Baca, 2013). In the time course of animal model trials, tonabersat was shown to inhibit neuronal-glial gap junctions in trigeminal ganglion (TGG), suggesting that this compound could prevent peripheral sensitization within the ganglion (Damodaram et al., 2008). Moreover, the signaling cascade that takes place between CSD induction and activation of the trigeminovascular system was recently elucidated and involved a transient opening of stressed neuronal pannexin1 (Panx1) channels (Karatas et al., 2013). The results of the clinical trials and pharmacokinetic studies indicate that tonabersat is more suitable as a daily prophylactic drug for migraine with aura attacks than in the acute treatment of migraine (Hauge et al., 2009; Silberstein, 2009).

The purpose of this review is to provide up-to-date information of our knowledge about the involvement of gap junction channels in migraine with aura pathophysiology and their emerging role as potential targets in prophylaxis treatment of this disease.

## Gap junction channels and hemichannels in the central nervous system

Gap junctions are specialized regions of the plasma membranes of adjacent cells, separated by an intercellular space (gap) of 2–3 nm, where transmembrane channels are clustered in microdomains. Gap junction channels assemble from the docking of two hemichannels, each of them originating from cells in contact. These channels connect the cytoplasms of the adjacent cells and allow the intercellular passage (gap-junctional intercellular communication; GJIC) of small molecules up to approximately 1 kDa (cAMP, IP_3_, metabolites, sugars, siRNA, and small peptides). Each hemichannel (or connexon) is an oligomerized hexamer of proteins called connexins (Cxs), surrounding an aqueous pore (1–1.5 nm diameter). To date, 20 members of the Cx family have been identified in mice and 21 in humans (Rackauskas et al., 2010). Cxs are expressed in all tissues except differentiated skeletal muscles, erythrocytes, mature sperm cells and are restricted to certain adult neuronal subpopulations (Söhl et al., 2004). Most cell types express multiple Cx isoforms, and therefore homotypic, heterotypic, and heteromeric gap junction channels may form between cells (Kumar and Gilula, 1996). Cxs have four hydrophobic membrane-spanning domains, two conserved extracellular domains (E1, E2) and three cytoplasmic domains such as both extremities and a loop between transmembrane domains 2 and 3. The C-terminal tail region, diverse in size and sequence, can be phosphorylated (except Cx26) and mediates interactions with partner proteins (Hervé et al., 2004; Sosinsky and Nicholson, 2005). A new protein family called Panxs (Panx1, 2, and 3) was recently discovered and classified as gap junction proteins due to their homology (25–33% identity) to the more than 25 innexins (Inxs) that have been identified as gap junction proteins of Invertebrates (Yen and Saier, 2007). However, Panxs bear no sequence homology with Chordate Cxs and current studies indicate that Panxs cannot form gap junction channels *in vivo*. In contrast, Panxs can form hemichannels that allow diffusion of ions and small molecules between intra and extracellular compartments. In Vertebrates, the largely overlapping distribution of Panxs and Cxs in tissues renders the molecular identity of the protein forming hemichannels at non-junctional membrane difficult to establish (Scemes, 2012). Gap junction channels play critical roles in many signaling processes, including coordinated cardiac and smooth muscle contraction, neuronal excitability, neurotransmitter release, insulin secretion, and others. The degree of GJIC is sensitive to a variety of stimuli, including growth factors, hormones, cytokines, neurotransmitters, lipophilic agents (alcohols, fatty acids, steroids, and others), changes in the level of intracellular Ca^2+^, pH, in transjunctional applied voltage and in phosphorylation/dephosphorylation processes (Budunova and Williams, 1994; Hervé and Sarrouilhe, 2005).

Cxs are largely represented in the CNS with eleven Cx subtypes (Cxs26, 29, 30, 32, 36, 37, 40, 43, 45, 46, 47). Some of them are expressed in the same cell type, forming intercellular channels with different structural combinations and properties whose physiological significance remains largely unknown (Nakase and Naus, 2004). GJIC is observed between astrocytes, oligodendrocytes, neurons (electrical synapses), microglia, ependymal cells as well as between neurons and astrocytes, and astrocytes and oligodendrocytes (Nakase and Naus, 2004; Orthmann-Murphy et al., 2007). In the nervous system, Cx43, Panx1, and Panx2 can form hemichannels at non-junctional membrane which physiological and pathophysiological roles remain to be determined (Kar et al., 2012). In the last decade, the link between inherited mutations in Cx genes, gap junction channel loss of function and human central (Cx47 and type 1 Pelizaeus-Merzbacher-like disease; Cx43 and oculodentodigital dysplasia) and peripheral (Cx32 and type 1 Charcot-Marie-Tooth disease) neuropathies was firmly established (Zoidl and Dermietzel, 2010; Abrams and Scherer, 2012). Changes in Cx expression and GJIC capacity were described in brain injuries and dysfunctions as inflammation, epilepsy, and neurodegenerative diseases (Rouach et al., 2002). Thereby, gap junction channels have progressively appeared as potential therapeutic targets in the treatment of a growing number of CNS diseases (Alldredge, 2008).

## Migraine with aura

### Pathophysiology of migraine with aura

**Figure 1:**
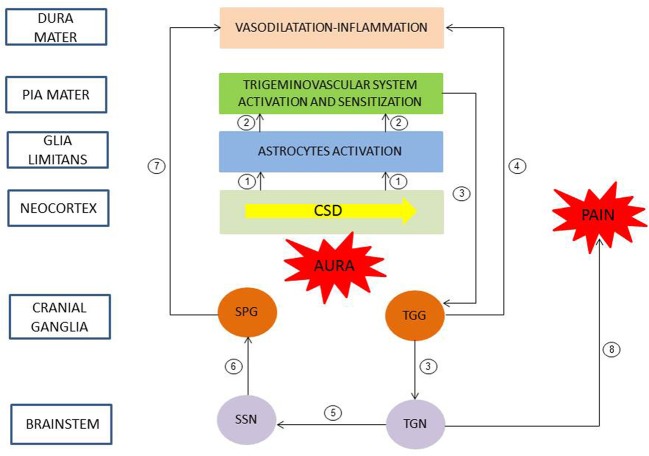
**Pathophysiology of migraine with aura.** A slow wave of neuronal and glial depolarization, cortical spreading depression (CSD), has been implicated in the mechanism of migraine aura. Moreover, CSD induces an opening of stressed neuronal Panx1 channels with subsequent release of proinflammatory mediators. These mediators activated astrocytes of the *glia limitans* leading to a continuous release of cytokines, prostanoids and nitric oxide to subarachnoid space (1). These molecules diffuse locally and depolarize and sensitize perivascular trigeminal terminals in *pia mater* (2, peripheral sensitization). In turn, the caudal portion of the trigeminal nucleus (TGN) of the brainstem is activated and sensitized (3, central sensitization). Collateral axons of activated neurons in the trigeminal ganglion (TGG) release proinflammatory peptides in the *dura mater*, inducing a sterile inflammatory reaction and then a headache (4). Moreover, a central trigeminal-parasympathetic reflex, originating from TGN and mediated through the superior salivary nucleus (SSN) of the brainstem and the sphenopalatine ganglia (SPG), produces vasodilatation of *dura mater* vessels (5–7). Pain perception is mediated by projections from the TGN to brain structures (8). Redrawn and modified after (Sarrouilhe and Dejean, 2012).

Progresses in understanding the pathophysiology of migraine was made in recent years (Goadsby et al., 2009a; Olesen et al., 2009). Even if the brain events in the development of migraine with aura are unclear in human, experimental data in murine corroborated by clinical observations allowed to propose a chronology of events linking aura and headache (Bolay et al., 2002). Neuroimaging and experimental studies suggested that CSD triggered migraine aura and was responsible of activation of the trigeminovascular system and possibly migraine headache (Figure 1). CSD is a transient disturbance in electroencephalographic activity characterized by slow wave of neuronal and glial depolarization, that self-propagates at a speed of 2–5 mm/s across the brain cortex or other brain areas (Charles and Baca, 2013). The depolarization phase of CSD is associated with a transient increase in cerebral blood flow. This depolarization is followed by a long-lasting suppression of neuronal activity accompanied by a prolonged decrease in cerebral blood flow. GJIC, and non-junctional Cx-containing hemichannels, have been proposed to be involved in CSD, and gap junction blockade would represent a possible therapeutic strategy (For review, see Costa et al., 2013). CSD causes large shifts in cortical extracellular ionic composition (H^+^, K^+^), pH, metabolites (nitric oxide (NO), arachidonic acid), and neurotransmitter (glutamate) concentrations (Bolay et al., 2002). These molecules diffuse locally, depolarize or sensitize perivascular nociceptive trigeminal afferents in *pia mater* (peripheral sensitization), which, in turn, sensitize and increase neural activity in the ipsilateral TGG and in the caudal portion of the trigeminal nucleus (TGN) in the brainstem (central sensitization). In the same time, collateral axons of activated neurons in the TGG release proinflammatory peptides (neurokinin A, CGRP, substance P) in the *dura mater*, leading to a local sterile inflammatory reaction (i.e., in the absence of infection) and then to headache. Moreover, a central trigeminal-parasympathetic reflex produces vasodilatation of *dura mater* (and *pia mater*) vessels. For this, the activation of the TGN, in turn, also induces a stimulation of the superior salivary nucleus (SSN) of the brainstem and perivascular parasympathetic efferents of the *dura mater* via the sphenopalatine ganglia (SPG). Postganglionic parasympathetic efferents release vasoactive molecules (vasoactive intestinal polypeptide, NO, acetylcholine) into the *dura mater*, promote vasodilatation and hyperemia and then could also explain autonomic symptoms that frequently occur during migraine attack. The perception of pain is mediated by rostral projections from the TGN to brain structures. It was proposed that in the attack of migraine with aura, nociception almost originated from pial perivascular trigeminal afferents with a later contribution from dural perivascular ones (Olesen et al., 2009).

The cellular mechanisms of initiation and propagation of CSD is still poorly understood. Astrocytes are highly interconnected by gap junctions and conduct signals in the form of intercellular Ca^2+^ waves. Transmission of Ca^2+^ waves may involve cell-to-cell diffusion of Ca^2+^-mobilizing second messengers through the gap junction channels between astrocytes, and the extracellular release of ATP through Cx43 and Panx1 hemichannels. ATP, in turn, can potentially act on the adjacent and /or distant astrocytic population, in an autocrine/paracrine fashion, thus amplifying the extent to which Ca^2+^ waves are transmitted (Scemes and Giaume, 2006). It was proposed that gap junction-mediated propagation of Ca^2+^ waves in astrocytes may represent the advancing front of CSD but that other factors, including K^+^ and glutamate, are necessary for sustained propagation (Martins-Ferreira et al., 2000). The *N*-methyl-D-aspartate (NMDA) receptor antagonist, MK-801, blocks CSD but, unlike the gap junction blocker carbenoxolone, does not inhibit transmission of Ca^2+^ waves (Peters et al., 2003). So, the advancing front of CSD may contribute to the trigger of the depolarization of surrounding neurons, leading to further release of glutamate and K^+^ into the extracellular environment. Interestingly, astrocytic Cx hemichannels can contribute to the release of glutamate in the extracellular space (Ye et al., 2003). Glutamate, in turn, through the NMDA receptor may then stimulate cytosolic Ca^2+^ oscillations in astrocytes, allowing CSD propagation (Martins-Ferreira et al., 2000). More recently, the signaling cascade that takes place between CSD induction and activation of the trigeminovascular system was elucidated in mice (Karatas et al., 2013). It is now evident that Panx1 is functionally linked to the purinergic P2X7 receptor and that the two proteins may also interact physically. Moreover, one or both of these proteins may be directly linked with the inflammasome, a multiprotein complex that mediates the innate inflammatory response (MacVicar and Thompson, 2009; Dahl and Keane, 2012). CSD induces a transient opening of stressed neuronal Panx1 channels with subsequent activation of the inflammasome, leading to a cleavage of caspase 1 to its proteolytic form and a release of pro-inflammatory agents [high-mobility group box 1 (HMGB1), interleukin-1β (IL-1β)], both of which take part in the initiation of the inflammatory response (Silverman et al., 2009; Karatas et al., 2013). Nuclear factor KappaB (NF-κB) is activated and translocated to the nucleus followed by cyclooxygenase-2 (COX2) and inducible nitric oxide synthase (iNOS) induction in astrocytes forming the *glia limitans.* The parenchymal inflammatory response leads to a continuous release of cytokines, prostanoids and NO to subarachnoid space that, in turn, promotes a prolonged activation of the perivascular nociceptive trigeminal afferents in *pia mater*. So, in contrast to mediators such as H^+^ and K^+^ that are transiently released during CSD, the parenchymal inflammatory response may provide continuous release of mediators required for sensitization of trigeminal nerve endings and lasting headache. The inhibition of Panx1 channel by carbenoxolone abolishes caspase 1 activation, NF-κB translocation and trigeminal activation. These results suggest that Panx1 channels may play a role as a reporter linking neuronal stress to inflammatory reaction (Karatas et al., 2013).

The genetic influences of migraine at the population level are largely unknown. Progress in genetic analysis has been largely restricted to rare monogenic subtypes of migraine as familial hemiplegic migraine. Recent genome-wide association study (GWAS) allowed the identification of various susceptibility loci for common forms of migraine. The minor allele of *rs1835740* on chromosome 8q22.1 was identified to be associated with migraine, and particularly in individuals with migraine with aura. *Rs1835740* is located between two genes involved in glutamate homeostasis, suggesting a possible link between the identified variant and the regulation of a neurotransmitter that has long been suspected to play a role in migraine pathophysiology (Antilla et al., 2010). The TRESK K2P potassium channel, encoded by the *KCNK18* gene (10q25.3) and known to be involved in pain pathways, has been linked to migraine with aura in a single family (Lafrenière et al., 2010). In another GWAS, three susceptibility loci for common forms of migraine (with or without aura) were identified and suggested a shared pathophysiology among migraine with or without aura. *TRPM8* (2q37.1) encodes a member of the transient receptor potential (TRP) superfamily of channels that is involved in neuropathic pain; *LRP1* (12q13.3) encodes a receptor that could modulate glutamatergic synaptic transmission; *PRDM16* (1q36.32) which potential role in migraine is unclear (Chasman et al., 2011).

Then, in genetically susceptible patients, mutations in these genes may reduce the threshold for CSD. Triggers of migraine attacks would induce CSD in such a hyper-excitable cortex by modifying the activity of ionic channels and then by altering ionic flow across cell membrane (Chakravarty, 2010). However, the respective roles and interaction of genetic and environmental factors in this multifactorial disorder remain to be determined.

### Anti-migraine therapy

**Figure 2:**
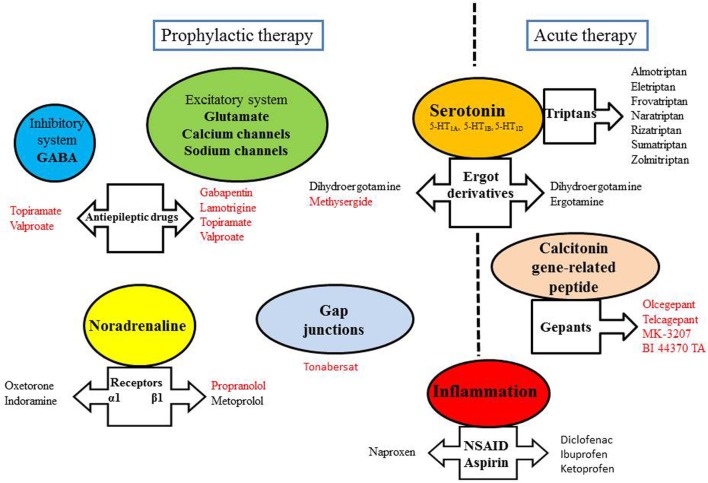
**Some targets of anti-migraine drugs.** Drugs written in red have been shown to exert an inhibitory effect on CSD.

Treatment for migraine is divided into acute and preventive approaches, aimed at stopping the evolving attack (relief of symptoms) or reducing the frequency of occurrence of attacks, respectively (Figure 2). The chronic treatment may last more than 1 year depending on the evolution of the disease. Acute treatment of vasodilatation and pain during attacks can control symptoms in 70% of attacks.

#### “Acute” therapy

Painful symptoms, arising from vasodilatation of cerebral vessels and inflammatory processes, require medication with powerful and fast action. The effectiveness of an acute therapy of migraine mainly depends on its earliness.

Some serotonergic agonists, which are antimigraine drugs with vasoconstrictive action, have high efficacy and are widespread prescribed. Ergot derivatives, which exert vasoconstrictive effect through the activation of 5HT_1A,1B,1D_ receptors, are also dopaminergic and adrenergic antagonists. Only two molecules are indicated in migraine attacks: ergotamine (exclusive use) and dihydroergotamine also used for prophylaxis. For all these drugs, there are oral, nasal and injectable forms, suitable for emergency treatment. Triptans cause vasoconstriction of the meningeal vessels by their agonist effect on the serotonin receptor 5-HT_1B_. They also induce an inhibition of pro-inflammatory neuropeptide release by trigeminal nerve endings through the activation of 5-HT_1D_ receptors. However, the effectiveness of triptan treatment during the aura phase remains controversial (D'Andrea et al., 2011). Indeed, it was proposed that the blood-brain barrier remains impermeable to triptans during the aura. Moreover, according to a study made with sumatriptan, the incorporation of 5-HT_1D_ receptors in the synaptic membrane would occur only after the onset of headache (Aurora et al., 2009). Because of their vasoconstrictive properties, triptans cannot be used in patients with vascular risk factors.

Migraine headache is associated with trigeminal nerve activation and CGRP release from the trigeminovascular system. CGRP is a vasodilatory neuropeptide and development of its antagonists is supported by the fact that they were highly effective in the treatment of migraine attacks (Edvinsson et al., 2012). Olcegepant, telcagepant, MK-3207 and BI 44370 TA constitute the new family of the gepants. They have displayed efficacy without vasoconstrictive effect, with fewer adverse effects and longer period than triptans (Costa et al., 2013). Moreover, the gepants have been reported to inhibit CSD (Figure 2).

The inflammatory reaction associated with the migraine is also a therapeutic target for some medications. Aspirin and NSAIDs such as ketoprofen, ibuprofen, diclofenac are effective on attacks of mild to moderate intensity. They act by inhibiting cyclooxygenase, and thus, prevent the synthesis of pro-inflammatory mediators, such as prostaglandins. Acetaminophen, despite widespread use, was poorly investigated as an anti-migraine drug. Anti-migraine agents can be addressed in combination to improve treatment compliance, efficacy or tolerability. For example, a triptan can be used 2 h after failure to NSAID treatment. The use of antiemetics (metoclopramide or domperidone) in acute migraine attacks is recommended because these drugs can improve the absorption of co-administered analgesics (Evers et al., 2009).

#### Migraine prophylaxis

Migraine prophylaxis is recommended when seizure frequency increases (more than two attacks per month) and/or intensity of the symptoms, as well as the duration of the aura. To reduce these parameters (frequency, intensity, or duration of crisis), a variety of drugs (sometimes off-label) acting through various mechanisms can be used.

Some of migraine drugs act through the serotonin receptors (pizotifen, methysergide, dihydroergotamine, oxetorone), calcium and sodium voltage-gated channels (flunarizine or anti-epileptics such as gabapentin, valproate, topiramate, and lamotrigine) or the glutamatergic neurotransmission (lamotrigine). Besides, topiramate and valproate are also GABA receptor agonists. Lamotrigine is an anti-epileptic drug that would specifically prevent migraine with aura (Lampl et al., 2005). A tricyclic antidepressant, amitriptyline, may prevent the secretion of neurotransmitters by modulating calcium channels (Wu et al., 2012). Moreover, the glutamatergic antagonism, as a protective action of neural response to CSD, is currently investigated (Olesen and Ashina, 2011). Adrenergic blockers can also be used to act on the vascular system (α-blockers: oxetorone, indoramine and antihypertensive β1 blockers: propranolol, metoprolol). Vascular reactivity is also modulated by inhibitors of angiotensin converting enzyme (lisinopril) and inhibitors of angiotensin II (telmisartan, candesartan). Naproxen, a NSAID, is used in prophylaxis as a modulator of inflammation. This is also the case of aspirin for treating migraines with aura. Finally, the mechanism of action of botulinum toxin (which inhibits the release of acetylcholine) has not been solved and the effectiveness of this toxin in prophylactic treatment appeared to be controversial in recent multicenter studies (Bekkelund and Alstadhaug, 2011; Marmura and Silberstein, 2011).

Drugs used in migraine prophylaxis (amitriptyline, methysergide, gabapentin, lamotrigine, topiramate, valproate, and propranolol) have been shown to experimentally suppress CSD susceptibility (Figure 2). Acute administration of these drugs was ineffective, only longer treatment duration produced CSD suppression (Costa et al., 2013). CSD-associated blood flow changes are experimentally modulated by α-amino-3-hydroxy-5-methyl-4-isoxazolepropionic acid (AMPA) and GABA receptors, suggesting that these receptors constitute targets of migraine therapy in inhibiting CSD (Holland et al., 2010). Furthermore, NR2B-containing NMDA receptors were shown to be key mediators of CSD, and as such, memantine- and some NR2B-selective antagonists may be useful therapeutic agents for the treatment of migraine with aura (Peeters et al., 2007).

In conclusion, if synapses, ionic channels, neurotransmitter receptors of neurons are numerous targets for such prophylactic treatment, the role of gap junction channels in CSD propagation, activation of the trigeminovascular system and peripheral (and central, see below) sensitization opens new perspectives of research.

## Gap junction channels as therapeutic targets

### Preclinical studies

Structure-activity relationship study of a new class of potential antimigraine agents identified tonabersat, a novel *cis* benzopyran, on the basis of its high efficacy at inhibiting neuronal hyperexcitability and trigeminal nerve stimulated neurogenic inflammation in rodent models (Chan et al., 1999). A release of NO occurs during the headache phase of a migraine attack and it is proposed that NO plays a pivotal role in depolarization and sensitization of perivascular nociceptive trigeminal afferents. Intraperitoneal administration of tonabersat (10 mg·kg^−1^) potently inhibited NO release following a cortical KCl stimulus in anaesthetized cats (Read et al., 2000). In a further work using the same anaesthetized cat model, tonabersat produces a marked dose-dependent inhibition of repetitive CSD and reduces CSD-induced repetitive episodes of pial artery vasodilatation (Smith et al., 2000). When compared with the serotoninergic agonist sumatriptan (5-HT_1D,1B_ agonist), tonabersat seemed to be devoid of adverse cardiovascular side-effects in isolated human tissues (MaassenVanDenBrink et al., 2000). In a study using repetitive diffusion-weighted MR imaging, the inhibitory effects of tonabersat on CSD event number and duration of activity was confirmed *in vivo* in the cat cerebral cortex. The drug also reduced significantly the magnitude of the cortical area involved in CSD episodes (Bradley et al., 2001). There is increasing evidence for a role of a *dura mater* neurovascular event, involving reflex activation of the parasympathetic nervous system, in migraine with aura attack. The effects of tonabersat were investigated in a model of trigeminal nerve parasympathetic neurovascular reflexes in anaesthetized cats, in which stimulation of the TGG produces an increase in carotid blood flow. Intravenous infusion of tonabersat (3.4 μmol·h^−1^) produced 4 h later a maximal inhibition of the neurovascular reflex (30% compared to control) whereas an intraduodenal administration (10 mg·kg^−1^) produced it (55% inhibition) 2 h later (Parsons et al., 2001). In spite of its broader spectrum of activity, tonabersat has been well tolerated and is devoided of CNS adverse effects, even at high doses. While clinical trials were started, a new mechanism of action was proposed for this drug (Damodaram et al., 2008). Within the TGG, cell bodies of afferent neurons are completely surrounded by several satellite glial cells that can influence neuronal activity by controlling their chemical environment. In rat, dye-coupling experiments demonstrated that neuronal-satellite cells can communicate via gap junction channels following neuronal activation in response to inflammatory stimuli rather than under normal basal conditions. These results suggested that neuronal-satellite glial cell signaling is involved in initiating and maintaining peripheral sensitization in the TGG and, thus, initiates migraine (Thalakoti et al., 2007). Under basal conditions, qPCR analysis of total RNA extracted from rat TGG allowed to detect mRNA of various Cxs (Cx26, Cx36, Cx40, and Cx43). Immunochemical analysis showed that Cx26 plaque formation between neurons and satellite cells was transiently increased in response to acute temporomandibular joint inflammation, and was sustained in chronic joint inflammation. Cx36 and Cx40 expression was shown to be only increased in neurons where they may form hemichannels. In contrast, Cx43 expression was not increased in TGG cells during acute or chronic joint inflammation. The temporomandibular joint model showed that inflammation duration was accompanied by differential Cx expression changes in TGG cells allowing increased GJIC between neurons and satellite cells (Garrett and Durham, 2008). Intraperitoneal administration of tonabersat (10 mg·kg^−1^) inhibited neuronal-satellite cell GJIC in TGG likely through a decrease of Cx26 expression since tonabersat treatment reduced Cx26 staining and number of junctional plaques (Damodaram et al., 2008). First, the results suggested that the high affinity and stereospecific binding site for tonabersat could be neuronal-glial cell gap junctions (Chan et al., 1999). Second, tonabersat should be able to prevent peripheral sensitization within the TGG.

Carbenoxolone, a potent blocker of both Cx43-made gap junctions and hemichannels and purinergic P2X7 receptor-associated Panx1 hemichannels completely suppressed central sensitization in an *in vivo* acute dental inflammation rat model and significantly attenuated it in an *in vivo* rat neuropathic pain model (Chiang et al., 2010; Wang et al., 2014). These results suggest that gap junctions and/or hemichannels play a critical role in mediating central sensitization in nociceptive neurons of the brainstem trigeminal *subnucleus caudalis* (often termed the medullary dorsal horn).

Probenecid, a well-established drug for the treatment of gout, is a powerful inhibitor of Panx1 hemichannels. Interestingly, Cx46 channels and the chimera Cx32E_1_43 channels (where the sequence of the first extracellular loop of Cx32 is replaced by that of Cx43) are not affected by probenecid (Silverman et al., 2008). In an *in vivo* rat model of migraine involving the systemic administration of the NO donor nitroglycerin, probenecid pre-treatment was shown to block the inflammatory process in the caudal TGN, also involved in the pathogenesis of migraine. Probenecid being also a non-selective inhibitor of multidrug resistance-associated protein and organic anion transporters, further studies are needed to determine the exact mechanism of the modulatory effect of this drug in trigeminal activation (Vamos et al., 2009).

Another study has shown that quinine, quinidine, and mefloquine are able to inhibit CSD in a rat neocortical slice model *in vitro* (Margineanu and Klitgaard, 2006). The alkaloids quinine, quinidine and the derivative mefloquine, commonly used as antimalarial drugs are known to reversibly and specifically abolish current through Cx36 channels (and with lesser potency through Cx50 channels) in transfected mammalian cells (Hervé and Sarrouilhe, 2005). The binding site for quinine is proposed to be intracellular, possibly within the pore of the gap junction channel (Srinivas et al., 2001). These results suggest an involvement of neuronal Cx36 channels in CSD propagation and might bear potential relevance for migraine with aura therapy (Margineanu and Klitgaard, 2006).

In conclusion, the preclinical studies identified tonabersat as a candidate for clinical evaluation of the treatment of migraine and gap junction channels as a potential therapeutic target.

### Clinical trials

A randomized, double-blind crossover, placebo-controlled study investigated the efficacy of tonabersat in the human experimental migraine model in which glyceryltrinitrate was used to induce migraine (Tvedskov et al., 2004). This two-center study (Denmark, UK) included 15 patients (18–55 years old), with 6–36 migraines without aura attacks per year. Patients were scheduled to have 2 study days separated by at least 11 days. On both study days, oral administration of tonabersat (40 mg) or placebo was followed 60 min after by glyceryltrinitrate infusion (0.5 μg/kg per min for 20 min in the cubital vein). The study was stopped prematurely after the unexpected observation of episodes of hypotension in 4 patients, which in two cases led to serious adverse events, suggesting a possible interaction between tonabersat and glyceryltrinitrate when the two compounds were concurrently administered. In the nine patients where comparison could be made, no significant preventive anti-migraine activity could be detected when tonabersat was compared with placebo. However, the number of treated patients in this study was too small to show any significance.

A second randomized, double-blind parallel group, placebo-controlled, multicenter (Denmark, Hungary, South Africa), phase II study further investigated tonabersat as a putative migraine prophylactic agent (Goadsby et al., 2009b). The clinical trial recruited patients (18–55 years old) experiencing between 2 and 6 migraine attacks (with or without aura) per month, for ≥ 1 year. The International Headache Society criteria for migraine with aura and migraine without aura were used to establish the diagnosis (Headache classification subcommittee of the International Headache Society, 2004). In total 160 patients were enrolled for a 4-week baseline observation period, after which 124 were randomized. The patients orally received tonabersat 20 mg daily (or matching placebo) for 2 weeks and 40 mg daily (or placebo) for further 10 weeks. 123 patients provided usable efficacy data, 58 in the tonabersat arm and 65 in the placebo arm. The study failed on its primary endpoint, the change in mean monthly migraine days from baseline to month 3 of the treatment period. In contrast, tonabersat had promising statistically significant effects on two predetermined secondary endpoints among ten, the change in the mean monthly consumption of rescue medication from the baseline period to month 3, and the overall 50% responder rate at 3 months. Unexpectedly, placebo responses were high for all endpoints and may have compromised the outcome of the study. However, the secondary endpoints data and the fact that tonabersat was very well tolerated support further exploration of this compound in a larger scale clinical trial.

A further single-center, randomized, double-blind parallel group, placebo-controlled phase IIb tonabersat evaluation in migraine prevention in the US (TEMPUS) involved approximately 500 patients with episodic migraine with or without aura. The TEMPUS study was significantly longer than the 3 months treatment period in the earlier phase IIa trial since the earlier trial showed that tonabersat was more effective toward the end of the 3 months period. The fact that tonabersat was very well tolerated in the previous study, allowed to explore the effects of increasing the dose (Goadsby et al., 2009b). Following a 4-week baseline observation period, the patients received either tonabersat (40 or 80 mg) or placebo on a once-daily basis. The 20-week study did not meet its primary endpoint of reducing the migraine attacks suffered by patients during the last 8 weeks of the 20-week treatment period compared to baseline period. A detailed analysis of the TEMPUS results has not been published (Peroutka, 2009).

An important step was taken forward with a fourth, single-center (Denmark), randomized, double-blind crossover, placebo-controlled phase II clinical trial. In contrast to the former, this study only included patients having migraine with aura exhibiting frequent aura attacks (Hauge et al., 2009). The study involved 39 patients (18–65 years old) who had at least 1 aura attack per month during the past 3 months. As previously reported, the patients suffering migraine with aura who were involved in the study had attacks with and without aura (Eriksen et al., 2004). Eight patients were excluded during the study and 31 completed the trial and were analyzed. Two treatment periods of 12 weeks (tonabersat and then placebo or placebo and then tonabersat) were separated by a washout period of 4 weeks with placebo. During each 12-week treatment period, tonabersat or matching placebo dose was increased from 20 to 40 mg at week 3. For the first primary endpoint, a significant reduction in the number of aura attacks, with or without headache, was observed during treatment with tonabersat compared with placebo. The median number of attacks of aura followed by headache was also significantly reduced. In contrast, the trial failed on its second primary endpoint, the reduction in migraine headache days with or without aura. The results showed that the gap junction antagonist, tonabersat, also known to inhibit CSD, has a preventive effect in migraine with aura but no efficacy on days with any type of migraine. These results are in agreement with previous studies using functional neuroimaging that have shown that cerebral spreading hypoperfusion, and probably CSD, are present only in migraine with aura (Sanchez del Rio and Alvarez Linera, 2004; Costa et al., 2013). Up to now, there is no direct evidence that CSD is the initiating mechanism in migraine without aura. The results of this crossover clinical trial do not support previous observation suggesting that an asymptomatic CSD is involved in attacks of migraine without aura (Woods et al., 1994). This small clinical trial requires confirmation in a larger randomized controlled preventive study in patients with migraine with aura.

However, what is the efficacy of tonabersat in the acute treatment of migraine? In 2009, two original articles described, in the same issue of Cephalalgia, the results of three multi-center phase II clinical trials that explored the ability of tonabersat to relieve the symptoms of migraine attacks. These randomized, double-blind parallel-group, placebo-controlled, multi-center, studies included patients (between 18 and 65 years old) suffering migraines with or without aura for at least 1 year before the trial, with 1–6 attacks each month. In the first study evaluating tonabersat as an acute treatment of migraine, the clinical trial was conducted in 53 centers in 12 countries (Dahlöf et al., 2009). A total of 693 patients suffering from migraine with or without aura were screened, among which 667 were enrolled and randomized to treatment in a 1:1:1:1 ratio. Patients received 20 or 40 mg of tonabersat, or 50 mg sumatriptan as a positive control, or placebo in a single oral dose at the onset of a moderate or severe attack. Among the 667 patients enrolled and randomized, 541 received allocated intervention and the results were analyzed for 406 of them. Tonabersat (20 or 40 mg) did not provide a significant advantage over placebo in headache relief determined 2 h after treatment (primary endpoint). Headache relief (rated on the International Headache Society scale), which is self-evaluated by the patient, is defined as a change in severity of the migraine symptoms from grade 2 (moderate) or 3 (severe) at pretreatment to grade 0 (none) or 1 (mild) 2 h after the treatment. Tonabersat had no effect on any secondary outcome variable, complete abolition of headache at 2 h and headache relief at 1 and 4 h, compared with placebo (Dahlöf et al., 2009). The efficacy of tonabersat in the acute treatment of migraine attacks has also been assessed in two other large clinical trials, one conducted in Europe, Australia, South Africa (named international study), and one in North America (USA, Canada) (Silberstein et al., 2009). In the International study, 549 patients were screened, among which 525 were enrolled and randomized and 441 received allocated intervention. In the North American study, 534 patients were screened, among which 506 were enrolled and randomized and 418 received allocated intervention. Patients took a single oral dose of tonabersat (15, 40, 80 mg in the International study; 25, 40, 80 mg in the North American study) or matching placebo at the onset of moderate or severe headache symptoms. The results were analyzed for 438 patients enrolled in the International study and 413 in the North American one. In the International study, tonabersat was significantly more efficient than placebo in relieving moderate to severe migraine pain. Significantly more patients who received tonabersat (40 mg) than those who received placebo experienced headache relief at 2 h (primary endpoint) or 4 h (secondary endpoint), or complete abolition of headache at 4 h (secondary endpoint). In the North American study, no significant benefits of tonabersat compared to placebo were observed. In these studies, previous triptan exposure appeared to affect the response to tonabersat. Moreover, pharmacokinetic analyses indicated that tonabersat was absorbed relatively slowly, and so required a long time to reach maximum plasma concentrations. The observed lack of efficacy may be a function of the slow absorption of tonabersat that could result in an insufficient therapeutic effect on acute migraine. Delayed absorption in patients with migraine has been previously reported during an attack and was proposed to be due to slower gastric emptying (Aurora et al., 2006; Silberstein et al., 2009). These pharmacokinetic characteristics indicate that tonabersat is more suitable as a daily prophylactic drug for migraine attacks (Silberstein, 2009).

## Conclusions and perspectives

Gap junction channels seem to be involved at different levels in the pathophysiology of migraine with aura. At a first level, GJIC and Cx-made hemichannels have been proposed to be involved in CSD propagation (Martins-Ferreira et al., 2000). The Cx36 channel blockers quinine, quinidine, and mefloquine are able to inhibit CSD in rats *in vitro*, suggesting an involvement of neuronal Cx36 channels in CSD propagation (Margineanu and Klitgaard, 2006). At a second level, the stressed neuronal Panx1 channels have been shown to be transiently opened in response to CSD propagation (Karatas et al., 2013). At a third level, in the TGG, gap junctions between neuronal and satellite glial cells seem to be involved in initiating and maintaining peripheral sensitization (Damodaram et al., 2008). And finally, at a fourth level, in the brainstem, it was suggested that gap junctions and/or hemichannels play a role in mediating central sensitization (Chiang et al., 2010).

Tonabersat, was selected for clinical trial on the basis of its inhibitory activity on CSD and neurogenic inflammation in animal models of migraine. Moreover, in preclinical studies, tonabersat was shown to inhibit GJIC between neurons and satellite glial cells within the TGG (Durham and Garrett, 2009). However, three randomized, placebo-controlled, clinical trials aimed at investigating the effectiveness of tonabersat as a preventive drug were negative (Tvedskov et al., 2004; Goadsby et al., 2009b; Peroutka, 2009). Moreover, conflicting results were obtained in dose ranging, placebo-controlled trials concerning its ability to relieve attacks (Silberstein et al., 2009). In contrast, in another randomized, placebo-controlled, clinical trial, tonabersat showed a preventive effect on attacks of migraine with aura but had no efficacy on non-aura attacks (Hauge et al., 2009). So, blocking CSD may represent a target for tonabersat in preventing migraine with aura. Diverse anti-migraine drugs have been shown to experimentally suppress CSD (For review, see Costa et al., 2013). Among them are the antiepileptic drugs (gabapentin, lamotrigine, topiramate, valproate), the β1 blocker propranolol, the tricyclic antidepressant amitryptiline, gepants and tonabersat. At a molecular level, these drugs exert their inhibitory effects on CSD, based on their mechanisms of action. Panx1 channel activity can be modulated by various mechanisms including activation via membrane depolarization, elevated extracellular K^+^, ionotropic receptors (including the NMDA glutamatergic receptor) and G protein-coupled receptors (Sandilos and Bayliss, 2012). It has been proposed that the advancing front of CSD may contribute to the trigger of the depolarization of the surrounding neurons, leading to further release of glutamate and K^+^ into the extracellular environment (Martins-Ferreira et al., 2000). Thus, inhibition of CSD propagation and, in turn, of release of glutamate and K^+^, could result in a reduced Panx1 channel activity. In the same way, inhibition of CSD, by reducing stressed neuronal Panx1 channel activity and, in turn, iNOS induction in astrocytes forming the *glia limitans*, could explain the potent inhibition of NO release observed following tonabersat pre-treatment in the CSD-anaesthetized cat model (Read et al., 2000; Karatas et al., 2013).

The extent to which migraine with aura and migraine without aura are different disorders has been the subject of much debate. Tonabersat, having a preventive effect in migraine with aura but no efficacy on non-aura attacks, suggests that an asymptomatic CSD is not involved in attacks of migraine without aura.

Preclinical studies and clinical trials also showed that tonabersat was well tolerated, with no effects on arterial blood pressure and heart rate. Moreover, the pharmacokinetic characteristics of tonabersat indicate that it is more suitable as a prophylactic therapy for migraine attacks. Comparing with the potential targets of currently utilized preventive drugs, tonabersat has a unique mechanism of action without vasoconstrictive adverse effect and might represent an interesting future possibility for the prophylaxis of this disease. It is estimated that about a third of migraineurs experiences aura. So, even if tonabersat benefits only to patients suffering migraine with aura, a substantial number of them could benefit from this drug. Moreover, the risk of ischemic stroke is doubled in migraine with aura, particularly in young women. The risk is greater for those with a higher frequency of migraine attacks (Kurth et al., 2012). Furthermore, MRI studies have shown an association between migraine with aura and a significantly increased risk of brain lesions (Kruit et al., 2010). The availability of a new therapeutic agent that could not only prevent the immediate discomfort associated with migraine but also potential long-term risks of repeated attacks, would be an important step forward. Migraine-preventive agents are still under-used while they could lower healthcare costs. The development of a preventive medication could reduce use of drugs for acute attacks and decreases visits to medical office and emergency departments.

Epilepsy and migraine are chronic neurological disorders with episodic manifestations that frequently occur together. Neuronal hyperexcitability is an underlying mechanistic similarity common to both disorders. Some antiepileptic drugs have a preventive effect in migraine without aura and possibly in migraine with aura (Haut et al., 2006). Across a number of preclinical studies, tonabersat also demonstrated an anti-seizure profile and is currently being evaluated in phase I studies as an investigational drug for treating epilepsy (Bialer et al., 2013).

The pathophysiology of migraine with aura involves GJIC between neurons and glial cells in the neocortex and TGG. Tonabersat treatment reduced Cx26 staining and number of junctional plaques in TGG cells. However, given the large number of Cxs expressed in nervous system, it is probable that other Cx proteins may also be regulated by tonabersat. Moreover, Panx1 channel activity at the plasma membrane being modulated by trafficking dynamics, a potential modulation of Panx1 trafficking by tonabersat requires experimental testing (Sandilos and Bayliss, 2012). Characterization of Cxs involved in the pathophysiology of migraine with aura and the development of models allowing their study are avenues to be explored in the future. This would allow the development of new therapeutic tools to treat efficiently and safely migraine with aura by suppressing or reducing specifically GJIC in cell subpopulations of the nervous system.

### Conflict of interest statement

The authors declare that the research was conducted in the absence of any commercial or financial relationships that could be construed as a potential conflict of interest.
